# Pro-Tumorigenic Phosphorylation of p120 Catenin in Renal and Breast Cancer

**DOI:** 10.1371/journal.pone.0129964

**Published:** 2015-06-11

**Authors:** Antonis Kourtidis, Masahiro Yanagisawa, Deborah Huveldt, John A. Copland, Panos Z. Anastasiadis

**Affiliations:** Department of Cancer Biology, Mayo Clinic, Jacksonville, Florida, United States of America; Northwestern University Feinberg School of Medicine, UNITED STATES

## Abstract

Altered protein expression and phosphorylation are common events during malignant transformation. These perturbations have been widely explored in the context of E-cadherin cell-cell adhesion complexes, which are central in the maintenance of the normal epithelial phenotype. A major component of these complexes is p120 catenin (p120), which binds and stabilizes E-cadherin to promote its adhesive and tumor suppressing function. However, p120 is also an essential mediator of pro-tumorigenic signals driven by oncogenes, such as Src, and can be phosphorylated at multiple sites. Although alterations in p120 expression have been extensively studied by immunohistochemistry (IHC) in the context of tumor progression, little is known about the status and role of p120 phosphorylation in cancer. Here we show that tyrosine and threonine phosphorylation of p120 in two sites, Y228 and T916, is elevated in renal and breast tumor tissue samples. We also show that tyrosine phosphorylation of p120 at its N-terminus, including at the Y228 site is required for its pro-tumorigenic potential. In contrast, phosphorylation of p120 at T916 does not affect this p120 function. However, phosphorylation of p120 at T916 interferes with epitope recognition of the most commonly used p120 antibody, namely pp120. As a result, this antibody selectively underrepresents p120 levels in tumor tissues, where p120 is phosphorylated. Overall, our data support a role of p120 phosphorylation as a marker and mediator of tumor transformation. Importantly, they also argue that the level and localization of p120 in human cancer tissues immunostained with pp120 needs to be re-evaluated.

## Introduction

p120 catenin (p120) was originally identified as a tyrosine phosphorylation substrate of the Src oncogene [1] but was subsequently recognized as a central player in cell-cell adhesion [2,3]. p120 interacts with E-cadherin (Ecad), the major cadherin member in epithelial tissues [2,4], and stabilizes it at the adherens junctions (AJs), by suppressing Ecad endocytosis [3,5–8]. Based on its ability to regulate E-cadherin stability, p120 is required for maintenance of the AJs and of proper formation of epithelial phenotypes [9]. AJ integrity is often compromised and progressively lost during tumor progression, contributing to increased rates of cell proliferation and migration [10,11]. Several studies have shown that p120 mis-localization or loss indeed results in pro-tumorigenic events [12–15]. However, recent studies have also shown that signaling events downstream of p120 and cadherins are crucial for the anchorage-independent growth of tumor cells, as well as for Src-mediated transformation [16–18].

Several p120 isoforms have been identified and named after the transcriptional start site used (1–4), and the alternatively spliced exons they express (A–D) ([19,20], reviewed in [21,22]). Changes in the ratio of p120 isoforms have been observed in epithelial *versus* mesenchymal cells [20,23,24]. In particular, the long isoform 1, which includes the N-terminal 323 amino acids, is responsible for pro-tumorigenic events, a function missing from isoform 4 that entirely lacks the N-terminal region [25].

In combination with the isoform expressed, p120 function is also regulated by phosphorylation. p120 can be phosphorylated in multiple serine, threonine and tyrosine residues [26–29]. Src family kinases phosphorylate p120 at a number of tyrosines (Y) within its N-terminus, including Y96, Y112, Y228, Y257, Y280, Y291, Y296, and Y302 [26]. EGFR can also phosphorylate p120 at Y228, without Src being the necessary intermediate [30]. Additionally, p120 is phosphorylated in several serine (S) and threonine (T) sites, including S122, S252, S268, S288, T310, S873, T910, some as a result of PKC activity (S268, S873) [27,31]. Notably, the S873 and T910 sites [27,32] of p120 isoform 1A correspond to S879 and T916 of full-length p120 used in the current database nomenclature, which includes the 6-amino acid long exon C (http://www.uniprot.org/uniprot/O60716; http://www.phosphosite.org/proteinAction.do?id=3241&showAllSites=true). Serine/threonine phosphorylation of p120 isoforms 1–3 controls E-cadherin dynamics at the cell membrane [28], while GSK3-dependent phosphorylation of p120 at T310 generates a front-to-rear gradient of p120 phosphorylation that regulates polarized trafficking of N-cadherin during collective cell migration [33]. Phosphorylation of p120 at several tyrosine and serine sites is inversely related to cadherin activation and adhesion strengthening [34,35]. Phosphorylation at S288 increases Kaiso binding and promotes lung cancer cell invasion [36]. Furthermore, Wnt signaling induces phosphorylation of p120 at S268 and S269, dissociating it from E-cadherin and subsequently promoting Kaiso sequestration and activation of downstream Wnt signaling events [37]. However, although in vitro evidence suggests a pro-tumorigenic role for p120 phosphorylation, the role of p120 phosphorylation in tumor progression is largely unknown and only a couple of studies have correlated p120 Y228 phosphorylation with progression of oral squamous cancer [38] and aggressiveness of glioblastoma [39].

Despite recent observations that p120 is required for the transformed and invasive phenotype of breast and renal cancer cells that have lost E-cadherin expression [17,25,40], for malignant transformation induced by oncogenic Src or Rac1 [18], and for invasion induced by HER2 [16], immunohistochemistry studies showed primarily mislocalization or loss of p120 in a variety of tumors [12,41–65]. In the majority of these studies, p120 expression was assessed using the pp120 monoclonal antibody (BD 610133), which was raised against the C-terminus end of p120 and recognizes all p120 isoforms [66]. Considering the inconcistencies of the *in vitro* and tissue expression findings, we have re-examined here the total expression of p120 and its phosphorylation levels in kidney and breast tumor samples, using a series of p120 specific antibodies previously described [32,66,67]. Our results show that p120 phosphorylation is elevated in the majority of tumor samples examined, consistent with our finding that a tyrosine phosphorylation-uncoupled p120 mutant is unable to induce transformed cell growth. Furthermore, the observation that cancer-specific phosphorylation of p120 at T916 interferes with epitope recognition of the pp120 antibody, provides an explanation for the discrepancies observed in previous studies.

## Results

### p120 phosphorylation at Y228 is elevated in tumor samples

**Fig 1:**
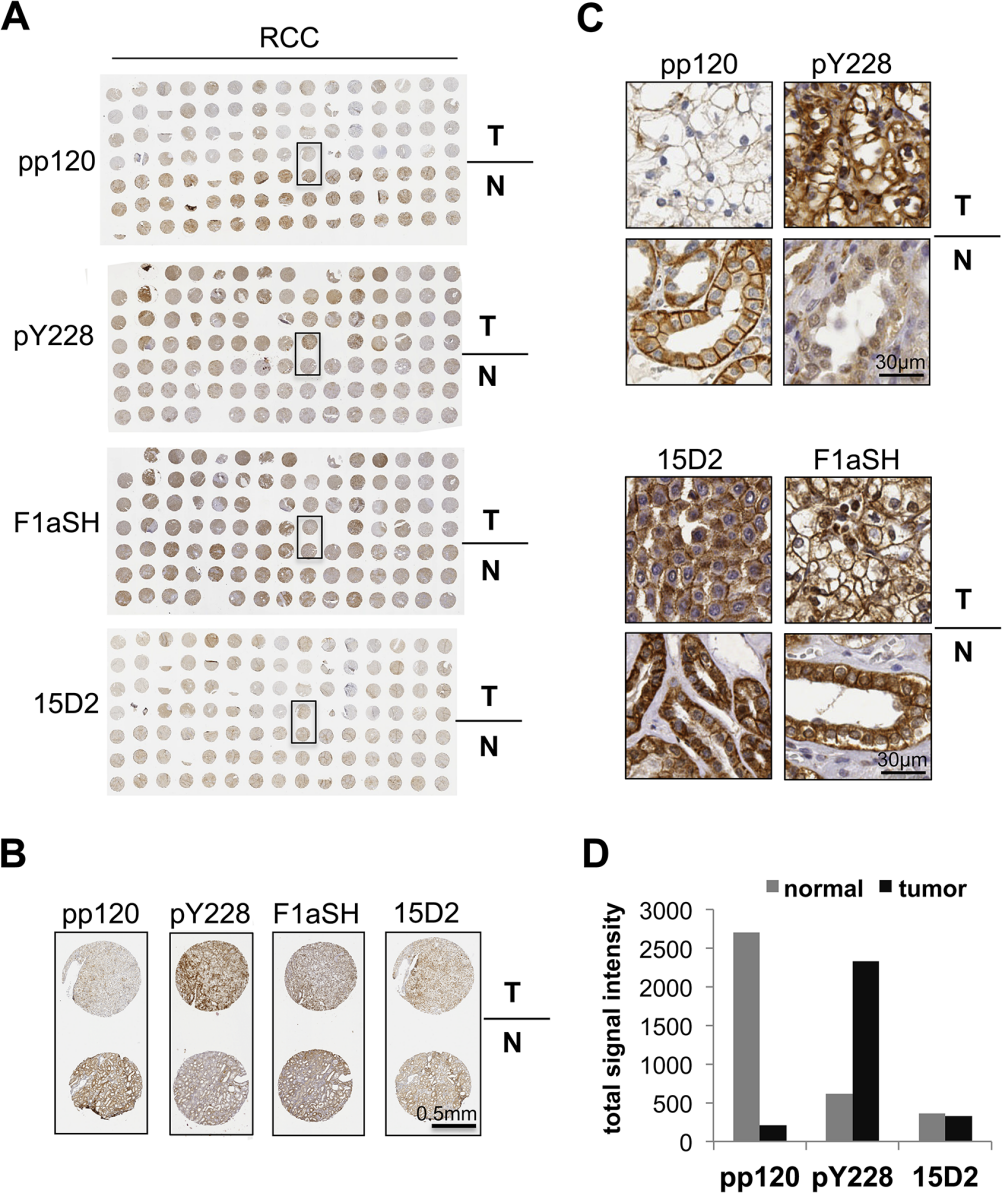
p120 phosphorylation at Y228 is elevated in RCC samples. Tissue microarrays (TMAs) of normal and tumor matched renal cell carcinoma (RCC) samples, stained by IHC for p120 using the pp120, pY228, 15D2 and F1aSH antibodies. The whole TMA stainings are shown in (**A**); a selected tumor-normal sample pair in (**B**); and details of the stainings of the selected pair in (**C**). Scale bars are shown on the bottom right in (B) and (C). (**D**) Quantitation of the signal of the TMA staining obtained by using pp120, pY228 and F1aSH; *n* = 15.

To evaluate expression and phosphorylation levels of p120 we initially performed IHC of tissue microarrays (TMAs) from stage 1 renal cell carcinoma (RCC) containing both tumor and patient-matched normal samples (Fig 1A). Total p120 levels were determined using the pp120, 15D2 and F1aSH antibodies [66], whereas an antibody recognizing tyrosine phosphorylated p120 at the Y228 site (pY228) [34] was used to assess the tyrosine phosphorylation status of p120. pp120, 15D2, and F1aSH IHC indicated that normal tissues were abundantly expressing p120 (Fig 1A–1C), whereas pY228 IHC showed low levels of Y228 phosphorylation. However, pY228 staining was significantly elevated in tumor tissues, compared to patient-matched normal samples (Fig 1A–1C). Interestingly, although total p120 was relatively unaltered in tumor samples compared to normal when using the 15D2 or F1aSH antibodies, staining with the pp120 antibody resulted in significantly less or absent p120 staining in tumor samples (Fig 1A–1C), Quantification of the signal obtained from pp120, Y228 and 15D2 IHC confirmed that although the 15D2 staining intensity is similar between normal and tissue samples, the pp120 signal is dramatically decreased and the Y228 signal is inversely increased in tumor samples (Fig 1D).

**Fig 2:**
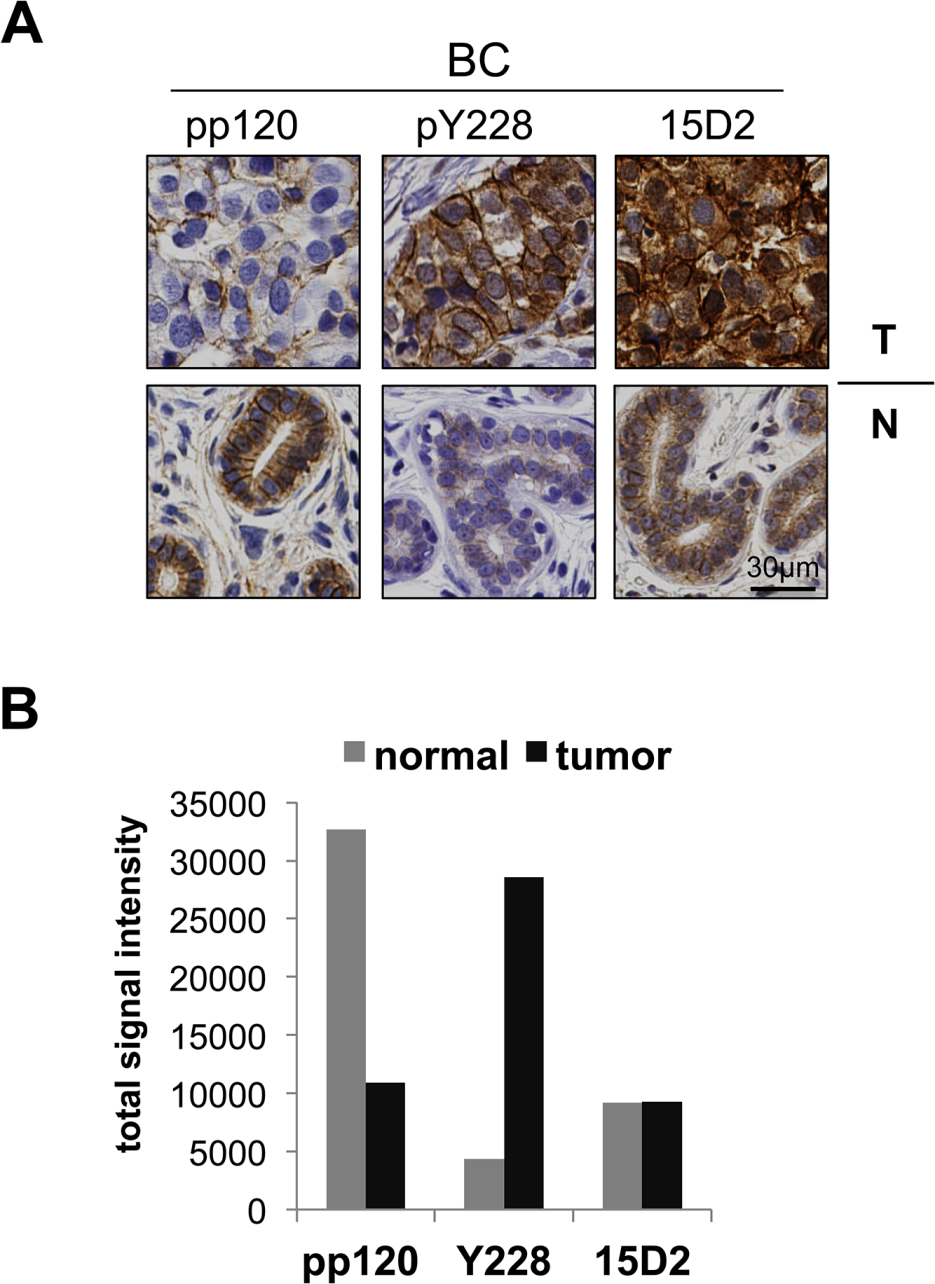
p120 phosphorylation at Y228 is elevated in BC tissues. (**A**) Breast normal and tumor (BC) samples stained by IHC for p120 using the pp120, pY228, and 15D2 antibodies. (**B**) Quantitation of the signal of the BC samples staining obtained by using pp120, pY228 and 15D2; *n* = 9.

To further examine the status of p120 phosphorylation and overall expression levels, we also stained a set of breast cancer (BC) samples using the pp120, pY228 and 15D2 antibodies (Fig 2A). Similar to RCC, pY228 was significantly upregulated in breast cancer samples compared to normal tissue, pp120 staining was markedly decreased in tumor tissues compared to normal, while 15D2 staining revealed abundant p120 staining in the same tumor tissues (Fig 2A and 2B). Therefore, the IHC results from both renal and breast cancer samples reveal a significant increase in p120 phosphorylation at the Y228 site, but also an unexpected inverse correlation between p120 phosphorylation and total p120 staining obtained with the commonly used pp120 antibody.

### The pp120 antibody binds the C-terminus of p120 around threonine 916

**Fig 3:**
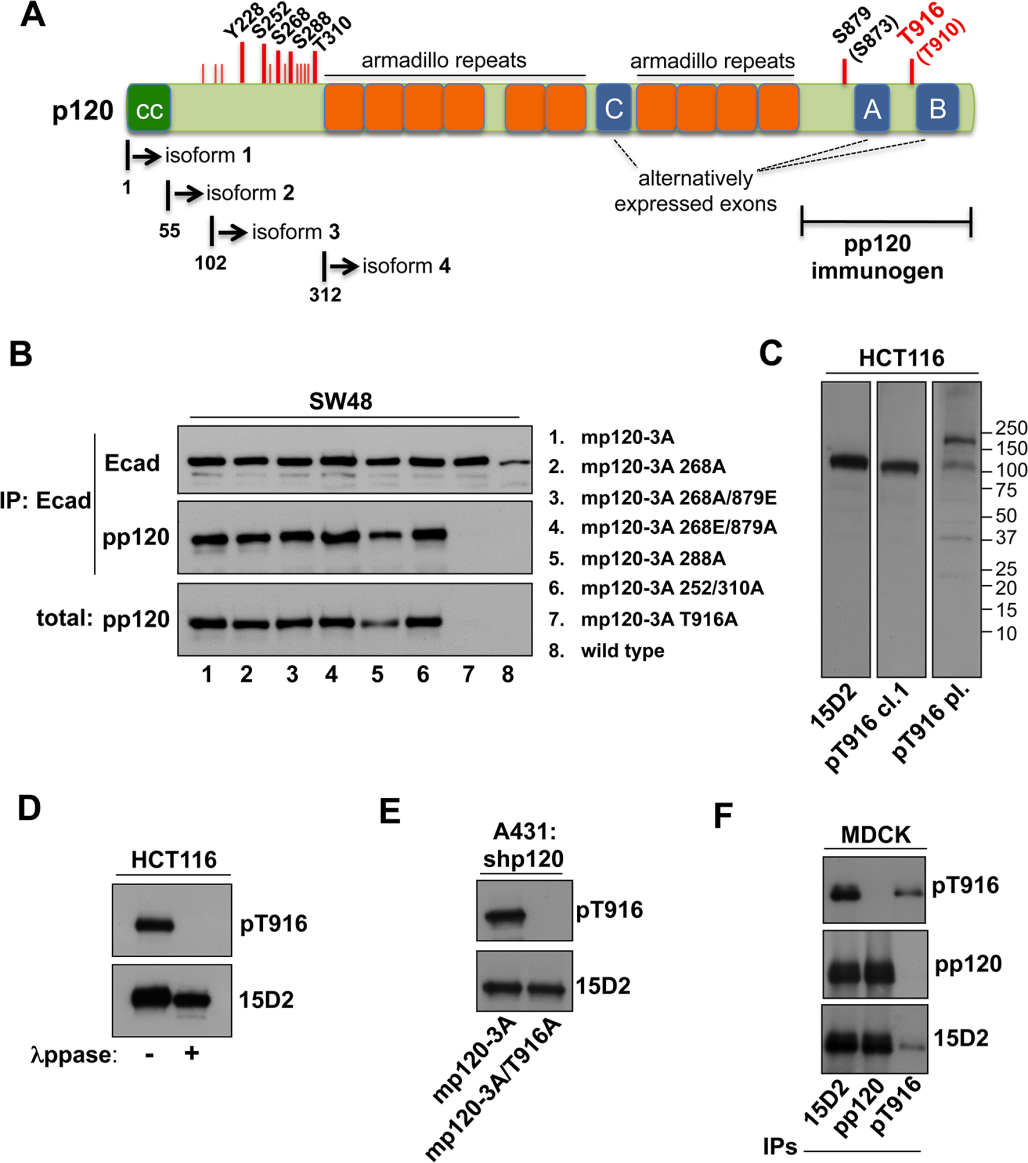
The pp120 antibody binds p120 around the T916 site and fails to recognize it, when it is phosphorylated. (**A**) Schematic of the p120 protein, depicting the different domains, the phosphorylation sites (in red lines), the different p120 isoforms and the region used as the pp120 immunogen. (**B**) E-cadherin (Ecad), and pp120 western blots of the p120-mutated SW48 colon cancer cells transfected with the constructs shown (1–7) or without (8), lysed and immunoprecipitated with E-cadherin. (**C**) Western blot of HCT116 cell lysates using either 15D2, a monoclonal T916 (cl.1) and a polyclonal T916 antibody (pl.). Molecular weights are shown on the right. (**D**) Western blot of HCT116 cell lysates treated with or without λ phosphatase and blotted for the monoclonal T916 (cl.1) and 15D2. (**E**) p120-depleted A431 cells were transfected with either murine wild type p120 isoform 3 (mp120-3A) or a construct with the T916 site mutated to alanine (mp120-3A T916A), and blotted for the monoclonal T916 (cl.1) and 15D2 antibodies. (**F**) Westen blot of MDCK lysates using pp120, 15D2 or T916 (cl.1) after immunoprecipitation with the same antibodies.

We then investigated the reasons behind the opposing observations in p120 abundance obtained with pp120 versus other p120 antibodies, or its correlation to p120 phosphorylation. The pp120 antibody was raised against the C-terminus of p120 and can recognize all p120 isoforms (Fig 3A) [66]. Since all tyrosine phosphorylation sites have been localized to the N-terminal domain, we focused on serine-threonine phosphorylation sites that could affect pp120 binding. Some of these serine-threonine sites reside at the C-terminus close to the pp120 target site, such as S879 and T916 (Fig 3A) [27,32,68]. To account for the underrepresentation of p120 levels in tumor tissues upon pp120 staining, we hypothesized that the epitope recognized by pp120 falls within or close to one of the phosphorylation sites of p120. To test this, we used the SW48 colon cancer cell line where the p120 gene is mutated and expresses very low amounts of only a truncated p120 form that lacks the C-terminus and does not bind E-cadherin (Ecad) [69]. As a result, Ecad is also expressed at very low levels in these cells, due to lack of p120-mediated stabilization [69]. By transient transfection, we introduced in SW48 cells either a construct expressing wild type murine p120 isoform 3A (mp120-3A; Fig 3A and 3B) or a series of mp120-3A constructs containing serine/threonine (S/T) to alanine (A) or glutamic acid (E) p120 mutations in the S252, S268, S288, T310, S879, and T916 phosphorylation sites (Fig 3A and 3B). We then performed Ecad immunoprecipitation of cell lysates containing the different constructs and blotted the precipitated fractions with either Ecad or pp120 (Fig 3B). Western blots showed that Ecad was successfully precipitated in all cases (Fig 3B). As expected, the levels of Ecad where significantly increased in all cells where exogenous p120 was introduced, including T916A (Fig 3B), indicating that the T916A p120 mutant was able to bind and stabilize Ecad. Interestingly, pp120 failed to recognize mp120-3A T916A in total cell lysates, although it recognized p120 in all other cases (Fig 3B). Consistent with this result, pp120 also failed to recognize mp120-3A T916A co-precipitation with Ecad, although it readily detected all other p120 mutants (Fig 3B). Therefore, the data suggest that pp120 selectively fails to recognize mp120-3A T916A, and raise the possibility that it recognizes an epitope that is on or around the T916 p120 phosphorylation site.

### A phospho-T916-specific antibody confirms that pp120 fails to recognize p120 phosphorylated at T916

We then used antibodies specifically developed to recognize phosphorylated p120 at T916. A polyclonal phospho-T916 antibody (T916 pl.) was previously described [32], whereas a monoclonal phospho-T916 is used here for the first time (T916 cl.1; BD 558398). Western blot of total cell lysates from HCT116 colon cancer cells showed that the monoclonal antibody produces a cleaner and more specific result in recognizing p120 compared to the polyclonal antibody (Fig 3C). To examine the phospho-specificity of the monoclonal pT916 antibody, we performed immunoprecipitation of HCT116 cells using the 15D2 antibody. The immunoprecipitates were split in two, and one part was treated with λ-phosphatase (Fig 3D). Analysis by Western blotting showed that the monoclonal pT916 antibody recognizes the phosphorylated but not the dephosphorylated p120, whereas 15D2 recognized p120 in both cases (Fig 3D). To further evaluate the specificity of the monoclonal pT916 antibody, we used A431 epidermal cancer cells that were depleted of endogenous p120 by retroviral shRNA expression (A431-shp120; Fig 3E). In these cells, we re-introduced either wild type murine mp120-3A or mp120-3A T916A (Fig 3E). Western blot analysis of A431 lysates expressing these constructs showed that the monoclonal pT916 antibody successfully recognized the wild type mp120-3A, but failed to recognize the phosphorylation-incompetent T916A p120 mutant (Fig 3E). 15D2 successfully recognized total p120 from cells bearing both constructs (Fig 3E). The above results collectively confirm that the monoclonal pT916 antibody is indeed specific for the phosphorylated T916 site of p120.

Subsequently, we used this monoclonal pT916 antibody to further evaluate whether pp120 recognizes a phosphorylated or a non-phosphorylated T916 epitope. We performed immunoprecipitation of canine MDCK cell lysates using the 15D2, pp120, or pT916 antibodies and subjected the isolated immunoprecipitates to western blot analysis using the same antibodies (Fig 3F). Western blots of pT916, pp120, and 15D2 confirmed successful pull-down of the respected target p120 populations in each immunoprecipitation (Fig 3F). Importantly, the 15D2 blot revealed successful precipitation of p120 in all three immunoprecipitates, including pT916 (Fig 3F). However, both pp120 and the pT916 antibodies failed to recognize co-precipitated p120 from each other (Fig 3F). This result, combined with the result we obtained in Fig 3B, indicates that pp120 recognizes only the non-phosphorylated T916 epitope of p120.

### T916 phosphorylation is increased in kidney tumor samples

**Fig 4:**
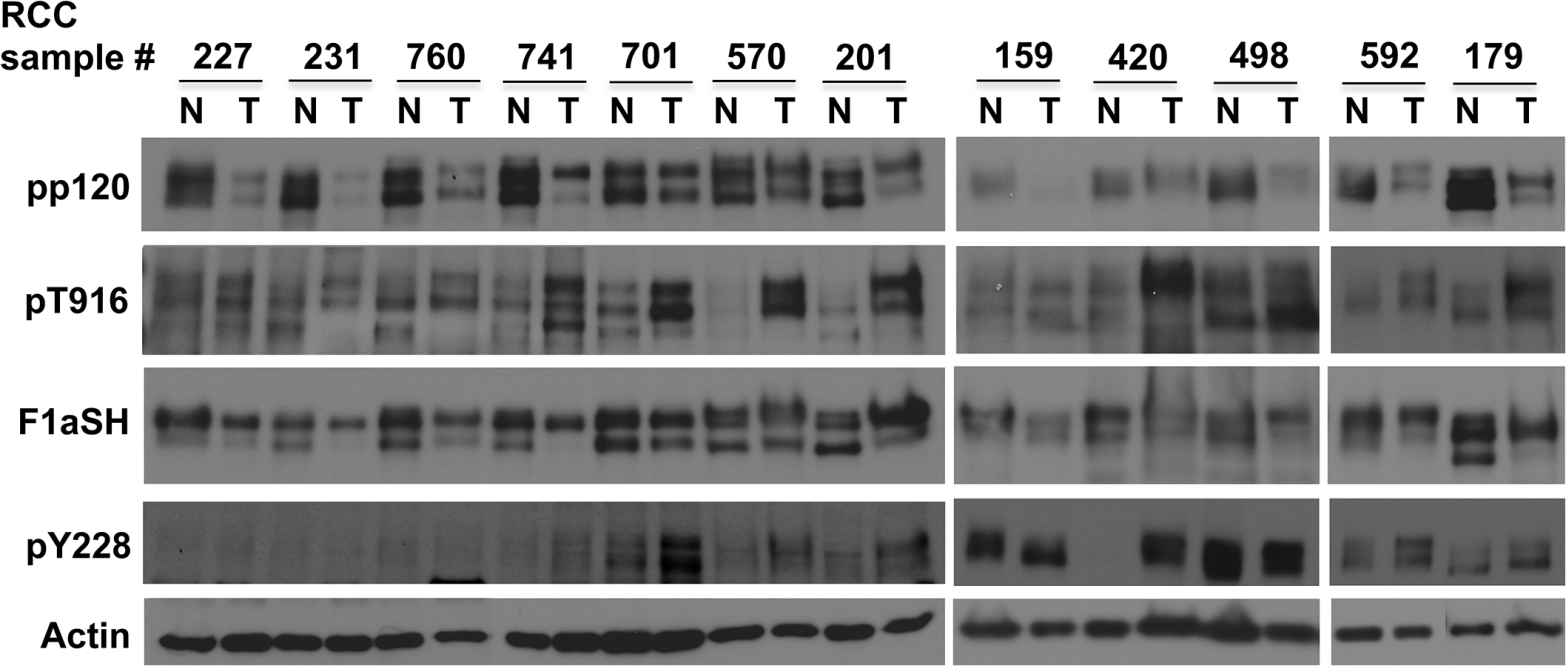
T916 phosphorylation is increased in RCC samples. Normal (N) and tumor (T) matched RCC samples were lysed and subjected to western blot for pp120, T916, Y228, F1aSH. Actin is the loading control.

We then used the newly characterized pT916 monoclonal antibody alongside the pY228 antibody to re-assess the phosphorylation status in kidney tumors. Our initial tests with the pT916 antibody indicated that in addition to junctional staining it produces a non-specific nuclear background by IHC (data not shown), preventing us from using this method to reliably evaluate T916 phosphorylation status in patient samples. For this reason, we isolated total protein from pairs of normal and tumor matched kidney samples from patients with stage 1 RCC and examined phosphorylation and total p120 expression by western blot (Fig 4). Results showed overall increased phosphorylation of p120 at T916 and confirmed the increased phosphorylation of Y228 in tumor tissues compared to normal patient-matched samples (Fig 4). This result is in agreement with phosphorylation data shown in Figs 1 and 2 and provides further evidence that p120 phosphorylation in multiple sites is a common event in cancer. Interestingly, western blot using the F1aSH antibody showed significant p120 expression in all tumor tissues tested, while the pp120 antibody detected significantly lower or absent p120 levels in 8 out of 12 tumor samples compared to their normal controls (Fig 4). These results further indicate that p120 is indeed present and phosphorylated in renal cancer tissues, and that the pp120 antibody selectively underrepresents total p120 levels due to p120 phosphorylation at T916.

### Tyrosine phosphorylation is critical for the transforming ability of p120

**Fig 5:**
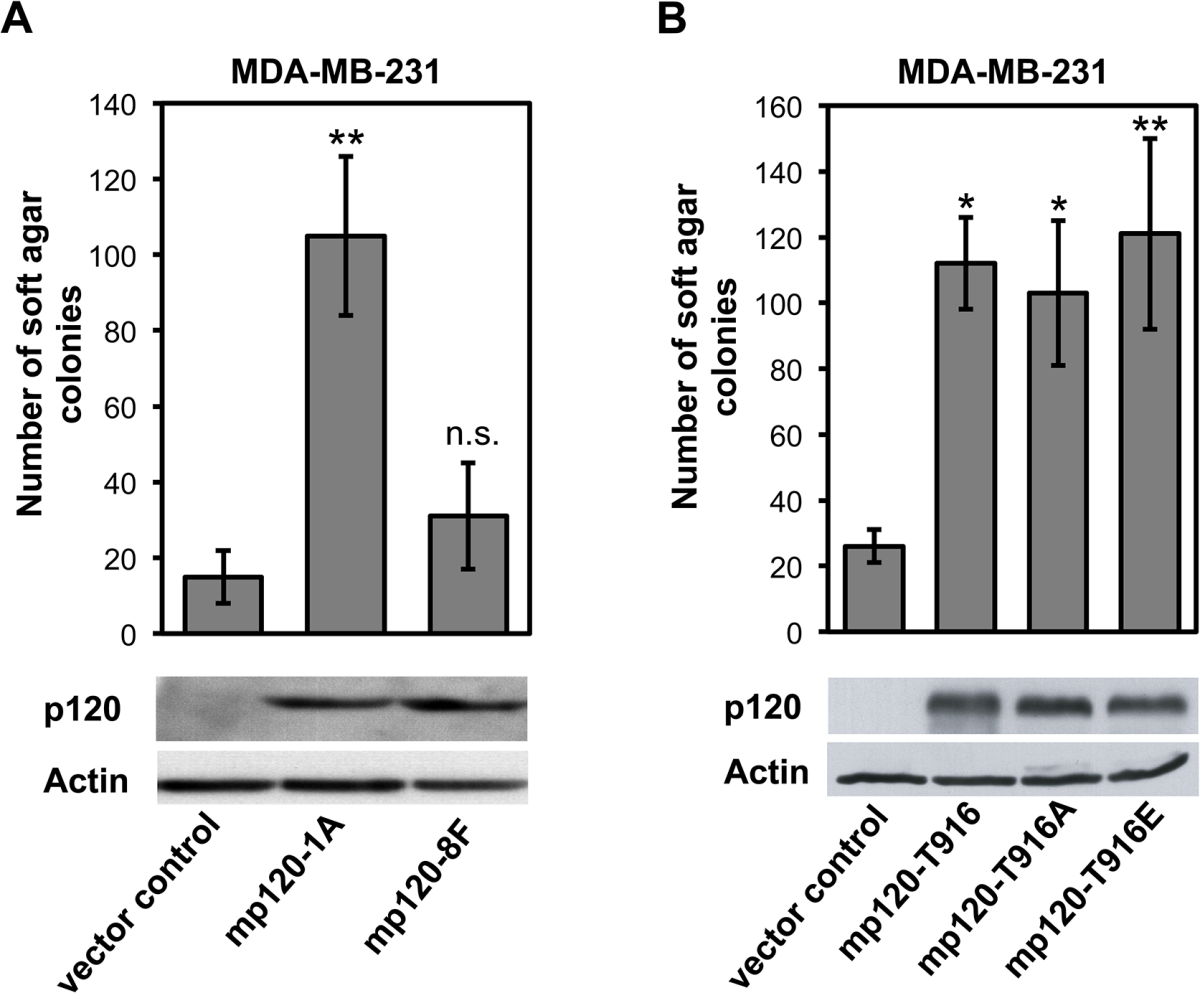
Phosphorylation at Y228 but not at T916 is critical for the transforming ability of p120. Breast cancer MDA-MB-231 cells were depleted of endogenous p120 and were transfected with: (**A**) vector control, full-length wild-type murine p120 isoform 1A (mp120-1A), or murine p120 isoform 1A where eight Src-targeted tyrosine phosphorylation sites, including Y228, were mutated to phenylalanine (mp120-8F); (**B**) vector control, murine wild type p120 (mp120-T916), or with murine p120 constructs where the T916 site was mutated to either alanine (mp120-T916A) or to glutamic acid (mp120-T916E). Cells in all cases were grown to soft agar and colonies were counted (mean ±SEM, *n* = 6; **p<0.01, *p<0.05, one-way ANOVA compared to vector control; n.s, non-significant). The western blots indicate expression of p120 using the 15D2 antibody in each case; Actin is the loading control.

Lastly, we examined the potential role of p120 phosphorylation in tumor progression. We have previously shown that p120 depletion reduces the anchorage independent growth (AIG) of MDA-MB-231 breast cancer cells, whereas ectopic expression of p120 re-instates it [17]. Here, we depleted MDA-MB-231 cells of p120 and re-introduced murine p120 constructs expressing: 1) wild type isoform 1A (mp120-1A) or a construct with eight Src-targeted tyrosine phosphorylation sites mutated to phenylalanine, including Y228 (mp120-8F)[26] (Fig 5A); 2) wild type T916 (mp120-T916), the phospho-incompetent alanine (mp120-T916A) or the phospho-mimetic glutamic acid (mp120-T916E) mutants (Fig 5B). We then examined the ability of the cells to grow on soft agar, a standard assay of AIG and of tumor transformation. Re-expression of the wild type p120 constructs resulted in a 4- to 5-fold increase in soft agar colony formation in both cases, confirming the previously described transforming ability of p120 (Fig 5A and 5B) [18,25,40]. However, the p120-8F mutant failed to recapitulate the effect, indicating that tyrosine phosphorylation at the N-terminus, including Y228, is required for the pro-tumorigenic function of p120 (Fig 5A). In contrast, the p120 constructs containing the mutated 916 sites resulted in similar increases in soft agar colony formation (Fig 5), suggesting that phosphorylation of p120 at this site is not an essential mediator of the transforming ability of p120.

## Discussion

Cell-cell adhesion proteins are fundamental in the maintenance of the normal epithelial phenotype, and play important roles in the process of malignant transformation when deregulated in cancer. p120 catenin is essential for epithelial cell adhesion, as a stabilizer of E-cadherin complexes [8,69], a function that accounts for its anti-tumorigenic properties. Consistent with this, a multitude of studies assessing the expression of p120 showed p120 mislocalization or loss in cancer tissues [16,18,67]. However, a number of recent observations suggest that p120 is indispensable for malignant cell growth and invasion *in vitro* [17,18,40]. p120 was shown to be essential for the anchorage-independent growth and increased migration and invasion of breast and renal cell lines that have lost E-cadherin expression [17,40]. Additionally, p120 was indispensable for the ability of HER2/ErbB2 to promote invasiveness in breast cancer cell lines [16]. Finally, cellular transformation by either oncogenic Src or Rac1 is p120-dependent [18]. These data suggest that p120 presence is required for tumor progression. To resolve this controversy we assessed p120 expression in a cohort of renal and breast cancer tissue samples using a number of p120 specific antibodies. As p120 can be phosphorylated at multiple sites by several pro-tumorigenic tyrosine kinases, including Src, EGFR, and Her2 [16,18,67], we also assessed p120 phosphorylation at Y228 as a marker of overall p120 tyrosine phosphorylation. Our results reconcile the previous contradictory observations, and show that p120 is generally present and phosphorylated in breast and renal tumor tissues. They also show that pp120, a monoclonal antibody widely used in previous p120 expression studies, underrepresents total p120 levels by failing to recognize p120 phosphorylated at T916, a cancer specific event.

In contrast to total p120 expression studies, information on the phosphorylation status of p120 is very limited [38,39]. Here, we provide evidence that phosphorylation of p120 in two sites, Y228 and T916, is significantly elevated in tumor vs normal breast and renal tissue samples. Importantly, p120 was originally isolated as a Src substrate [70], is phosphorylated by Src at eight N-terminal tyrosine sites, including Y228 [68], and is required for Src mediated transformation [18]. We show here for the first time that not only p120 phosphorylation at Y228 is elevated in cancer, but that tyrosine phosphorylation is required for the transforming ability of p120 (Fig 5). The data suggest that p120 phosphorylation at Y228 may serve as an important prognostic marker for tumor agressiveness.

In parallel, we show that although phosphorylation of the T916 site is not required for the pro-tumorigenic function of p120, it has implications in total p120 detection, when using the commercially available pp120 antibody. Therefore, results obtained in previous studies using pp120 should be re-evaluated, as p120 phosphorylation at T916 could lead to underrepresentation of p120 levels in these tissues. For example, if one relies on pp120 staining, p120 expression is severely downregulated in RCC as shown in Fig 1. However, this is a false conclusion, when one considers p120 phosphorylation and total p120 levels examined by other p120 specific antibodies. The subcellular localization of p120 should also be re-evaluated in these studies, as it is currently unclear how the T916 phosphorylation status affects p120 subcellular localization.

It is important to note here that loss of p120 expression may still be important for the progression of certain tumor types by destabilizing E-cadherin/catenin complexes. This is clearly illustrated by the lack of p120 expression in SW48 cells and the resulting loss of E-cadherin mediated cell-cell adhesion [69]. Nonetheless, our data indicate that loss of p120 expression in either renal or breast cancer is not the prevailing observation. This conclusion is in agreement with the *in vitro* data on the role of endogenous p120 in promoting the progression of both renal and breast cancer.

## Materials and Methods

### Antibodies—Constructs

The pp120, 15D2, F1aSH antibodies were previously described [66] and are also commercially available: pp120, BD Transduction Laboratories, 610133; 15D2, Life Technologies, 33–9600. The phospho-specific p120 antibodies Y228 and T916 were also previously described [32,67] and are commercially available: Y228, BD Transduction Laboratories, 612536; T916, BD Pharmingen, 558398. Ecad is from BD Transduction Laboratories (610182) and Actin from Cell Signaling (4967). The pRS (pRetro-SUPER) control and p120-targeting shRNA retroviral vectors were previously described [5]. The mp120-3A, mp120-3A 268A, mp120-3A 268A/879E, mp120-3A, 268E/879A, mp120-3A 288A, mp120-3A 252/310A, mp120-3A T916A, and mp120-3A T916E constructs are based on the retroviral LZRS vector and were previously described [32]. The mp120-1A and mp120-8F constructs were previously described [68].

### Cell lines, transfection—infection

MDCK canine kidney epithelial cells, MDA-MB-231 breast carcinoma cells, A431 epidermoid carcinoma cells, and HCT116 colon carcinoma cells were cultured in DMEM (Cellgro) supplemented with heat-inactivated 10% FBS (Mediatech). SW48 cells were grown in DME/F-12 with 10% FBS and 1%L-glutamine. All lines were obtained from ATCC. Cells were transfected using Lipofectamine 2000 (Invitrogen), according to the manufacturer’s protocol. LZRS and pRS retroviruses and infections were prepared and performed as described previously [17,27].

### Tissue collection—Ethics statement

Renal tissue samples were initially collected for the Mayo Clinic Renal Tissue Registry under protocol 14–03, with the approval of the Mayo Clinic Institutional Review Board. Similarly, breast tissue samples were initially collected for the Breast Center Blood and Tissue Bank with the approval of the Mayo Clinic Institutional Review Board protocol 09–001909. Written informed consent for the use of these tissues in research was obtained from all participants to these Registries. Both renal and breast tissue samples used in this study were obtained from these two registries under the Mayo Clinic Institutional Review Board protocol 675–05: "RCC metastasis: Effect of smoking, targeted therapy, and development of novel prognostic markers". All unique patient identifiers and confidential data were removed and tissue samples were deidentified. The Mayo Clinic Institutional Review Board assessed the 675–05 protocol as minimal risk and waived the need for further consent. All data was analyzed anonymously.

### Immunohistochemistry

Paraffin-embedded renal tissue microarrays (TMAs) containing renal tumor and matched normal samples were stained. Briefly, slides were placed in xylene and rehydrated in a graded ethanol series, rinsed in water, subjected to heat antigen retrieval according to the manufacturer’s instructions (Dako), incubated with primary antibody and finally with either anti-rabbit (for F1aSH) of anti-mouse (for pp120, 15D2, Y228) labeled polymer horseradish peroxidase (Dako). Dilutions for primary antibodies: pp120, 1: 800; Y228, 1:200; F1aSH, 1: 800; 15D2, 1:800. Slides were scanned using Aperio ScanScope XT and viewed and quantified using Aperio ImageScope v11.1.2.752.

### Tissue protein extraction

Fresh frozen renal tissues were used for protein extraction. Briefly, tissues were weighted and then lysed in 20-fold excess of the following lysis buffer: 2% SDS, 4M Urea, 62.5 mM Tris-HCl pH 6.8, 1mM EDTA, 60 mM β-mercaptoethanol. Lysis was performed with two cycles of sonication on ice, 10 sec each. Lysed tissues were cleared by tabletop centrifugation at full speed, for 5 min at 4°C. Supernatants were then passed through a 29g needle to decrease viscosity and spun again, as above. Supernatants were transferred to clean tubes and stored at -20°C. Protein quantification of the acquired samples was performed using the RC-DC assay (Bio-Rad). Finally, samples were mixed with 10x Laemmli sample buffer (LSB) to obtain a final 2x LSB, boiled for 5 min and used for Western blotting.

### Immunoprecipitation

Cells were washed once with PBS, and then lysed in RIPA buffer (50 mM Tris, pH 7.4, 150 mM NaCl, 1% NP-40, 0.5% deoxycholic acid, 0.1% SDS) containing protease and (protease inhibitor cocktail III, RPI) and phosphatase inhibitors (Pierce). Lysates were cleared by centrifugation and total protein levels were measured using the BCA assay (Pierce). Where whole cell lysates were used, they were mixed with 10x Laemmli sample buffer (LSB) to a final 2x LSB, boiled for 5 min and used directly for Western blotting. For immunoprecipitation, primary antibodies were added to the supernatant and incubated for 1 h at 4°C with end-over-end rotation. Protein G-Sepharose beads were then added to lysates for another hour incubation at 4°C. Immunoprecipitates were washed four times with lysis buffer, re-suspended in 2x LSB, and boiled.

### Western blot

Denatured proteins were separated by SDS–polyacrylamide gel electrophoresis (PAGE) and transferred to nitrocellulose membrane (Biorad). Nonspecific binding to membranes was blocked for 10 min with 3% nonfat milk in Tween-Tris-Buffered Saline (T-TBS; 10 mM Tris pH 7.4, 150 mM NaCl, 0.1% Tween). Blots were incubated overnight in primary antibody in blocking solution at 4°C, washed 3x in T-TBS and subsequently incubated for 1 h at room temperature in secondary antibody (Jackson Immunoresearch, Inc.) diluted in blocking solution. For Western blotting, primary antibodies were used at the following dilutions: pp120, 1:2000; 15D2, 1:100; F1aSH, 1:2000; T916, 1:500; Y228, 1:500; Ecad, 1:2000; Actin, 1:2000. Secondary antibodies were used at 1:2000 dilutions. Blots were developed by enhanced chemiluminescence (ECL, Amersham).

### λ-phosphatase treatment

After immunoprecipitation, the p120-containing bead complex was washed three times with RIPA lysis buffer without phosphatase inhibitors, followed by two times with TBS (pH 7.4). A 50-μl λ-phosphatase reaction (protein sample, 50 mM Tris-HCl, pH 7.5, 0.1 mM Na_2_EDTA, 5 mM EDTA, 2 mM MnCl2, 200 units of λ-phosphatase and dH2O) was carried out at 30°C for 30 min. An identical control reaction was carried out in the absence of λ-phosphatase. The beads were then spun down and re-suspended in 2xLSB. Protein was denatured and loaded on SDS-PAGE gel for Western blotting.

### Soft agar assay

The assay was performed in 6-well plates (Costar 3516). Briefly, wells in the plates were first covered with a layer of 0.75% agarose, prepared by mixing 1.5% of sterile agarose (Seakem) in a 1:1 ratio with 2x concentrated culture medium made freshly from powder DMEM (Invitrogen) and double addition of FBS. The base layer was left to solidify for 30 min and cells were then trypsinized, counted and re-suspended in 2x medium. The cell suspension was mixed in a 1:1 ratio with 0.7% sterile agarose to make a final layer of 0.35% agarose, which was added on top of the base layer. 5x10^3^ cells were seeded per well. The top layer was left to solidify for another 30 min and was covered with regular (1x) culture medium. Cultures were maintained for 3–4 weeks with medium renewal on top of each agar layer every three days, until visible colonies were formed. At the end of the experiment, plates were stained with 0.02% Crystal violet (Fisher) prepared in 20% Ethanol and PBS, washed three times with distilled H_2_O, scanned and counted for colonies. Statistics were performed using GraphPad Prism 6.
